# Structural mechanism for the arginine sensing and regulation of CASTOR1 in the mTORC1 signaling pathway

**DOI:** 10.1038/celldisc.2016.51

**Published:** 2016-12-27

**Authors:** Zhongchao Gai, Qian Wang, Can Yang, Lei Wang, Wei Deng, Geng Wu

**Affiliations:** 1 State Key Laboratory of Microbial Metabolism, School of Life Sciences & Biotechnology, Shanghai Jiao Tong University, Shanghai, China; 2 National Center for Protein Science Shanghai, Shanghai, China

**Keywords:** mTORC1 signaling pathway, arginine, CASTOR1, GATOR2, GATOR1, crystal structure

## Abstract

The mTOR complex I (mTORC1) signaling pathway controls many metabolic processes and is regulated by amino acid signals, especially arginine. CASTOR1 has been identified as the cytosolic arginine sensor for the mTORC1 pathway, but the molecular mechanism of how it senses arginine is elusive. Here, by determining the crystal structure of human CASTOR1 in complex with arginine, we found that an exquisitely tailored pocket, carved between the NTD and the CTD domains of CASTOR1, is employed to recognize arginine. Mutation of critical residues in this pocket abolished or diminished arginine binding. By comparison with structurally similar aspartate kinases, a surface patch of CASTOR1-NTD on the opposite side of the arginine-binding site was identified to mediate direct physical interaction with its downstream effector GATOR2, via GATOR2 subunit Mios. Mutation of this surface patch disrupted CASTOR1’s recognition and inhibition of GATOR2, revealed by *in vitro* pull-down assay. Normal mode (NM) analysis revealed an ‘open’-to-‘closed’ conformational change for CASTOR1, which is correlated to the switching between the exposing and concealing of its GATOR2-binding residues, and is most likely related to arginine binding. Interestingly, the GATOR2-binding sites on the two protomers of CASTOR1 dimer face the same direction, which prompted us to propose a model for how dimerization of CASTOR1 relieves the inhibition of GATOR1 by GATOR2. Our study thus provides a thorough analysis on how CASTOR1 recognizes arginine, and describes a possible mechanism of how arginine binding induces the inter-domain movement of CASTOR1 to affect its association with GATOR2.

## Introduction

The target of rapamycin (TOR) pathway (known as mammalian TOR, mTOR, pathway in mammals) is a central signal transduction pathway regulating many metabolic processes such as protein translation, lipid synthesis, autophagy, and so on in a vast variety of organisms from yeast to human. Aberrant regulation of the mTOR pathway in human predisposes to many diseases including cancer, diabetes, and autoimmune diseases [1, 2]. The mTOR pathway is under stringent regulation by various upstream nutrient signals such as amino acids [3, 4]. Amino acid availability determines the nucleotide bound state of the heterodimeric Rag GTPases consisting of RagA or RagB in complex with RagC or RagD [5]. Sufficient supply of amino acids promotes GTP-binding for RagA/RagB and GTP hydrolysis to GDP for RagC/RagD, which in turn recruits the mTOR complex I (mTORC1) (consisting of mTOR, Raptor, and mLST8) to its lysosomal anchor, the pentameric Ragulator complex [6, 7]. The mTORC1 complex is then activated by the lysosome-located small GTPase Rheb [8, 9], and phosphorylates a vast spectrum of downstream targets including ribosomal S6 kinase 1 (S6K1) and eIF-4E-binding protein 1 (4E-BP1).

As a key player for transmitting amino acid signals, the Rag proteins are regulated by multiple distinct factors, such as the Ragulator complex which serves as a guanine nucleotide exchange factor (GEF) for RagA/RagB [7], the FLCN/FNIP2 complex, which is a GTPase-activating protein (GAP) for RagC/RagD [10, 11]. The trimeric GATOR1 complex, comprising of DEPDC5, NPRL2, and NPRL3, functions as a GAP for RagA and RagB and inhibits their activities [12]. The GATOR1 complex is further regulated by its upstream inhibitor, the GATOR2 complex, which consists of five subunits: Mios, WDR59, WDR24, Seh1L, and Sec13 [12].

Recently, the GATOR2 complex has been found to have a critical role in mediating amino acids signals, especially leucine and arginine, to the mTORC1 pathway. The sestrin protein was identified as a cytosolic leucine sensor, and interacts with GATOR2 to inhibit mTORC1 signaling when leucine is scarce [13–16]. On the other hand, the CASTOR1 homodimer and the CASTOR1-CASTOR2 heterodimer function as a cytosolic arginine sensor, and negatively regulate mTORC1 activity by binding to and inhibiting GATOR2 [17]. In the absence of arginine, CASTOR1 binds to GATOR2 and inhibits mTORC1 signaling; whereas in the presence of arginine, CASTOR1 interacts with arginine and no longer associates with GATOR2. Both CASTOR1 and CASTOR2 were found to contain two putative Aspartate kinase, Chorismate mutase, and TyrA (ACT) domains which function to recognize small ligands such as amino acids and nucleotides [18–24]. Besides Sestrin and CASTOR proteins, the membrane transporter SLC38A9 was reported to be a lysosomal arginine sensor, and transmit the arginine signal to mTORC1 by binding to the Ragulator complex [25–27].

In this study, we determined the crystal structure of human CASTOR1 in complex with a tightly bound arginine. Our structure shows that CASTOR1 dimerizes mainly through juxtaposition of its *α*1 and *α*5 helices. An exquisitely tailored pocket, carved between the N-terminal domain (NTD) and the C-terminal domain (CTD) of CASTOR1, is employed to recognize arginine, and mutation of critical residues in this pocket abolished or diminished arginine-binding. Comparison with a prokaryotic aspartate kinase hinted that a surface patch on CASTOR1-NTD was involved in recognizing and activating GATOR2 (in particular, its subunit Mios only), which was confirmed by our pull-down assay using purified proteins. NM analysis suggested that a possible slicing motion between CASTOR1-NTD and -CTD might lead to exposure of its GATOR2-binding residues, thus regulating its association with GATOR2. Most likely, this breathing motion of CASTOR1 is correlated with arginine-binding. Finally, we proposed a model of how dimerization of CASTOR1 relieves the inhibition of GATOR1 by GATOR2.

## Results

### Overall structure of CASTOR1

**Figure 1:**
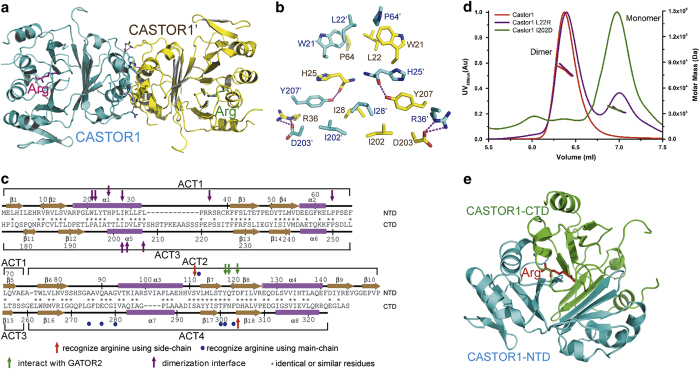
Structure of human CASTOR1. (**a**) Overall structure of the human CASTOR1 dimer. The two CASTOR1 protomers are colored in cyan and yellow, respectively; and the two bound arginine molecules are colored in magenta and green, respectively. (**b**) The dimerization interface of CASTOR1. The residues belonging to the two CASTOR1 protomers are colored in cyan and yellow, respectively. Hydrogen bonds are shown as magenta dashed lines. (**c**) Residues of the NTD and the CTD of CASTOR1 are aligned, with their secondary structures shown. Residues recognizing arginine using side-chains or main-chains are marked by red arrows or blue circles, respectively; while residues interacting with GATOR2 and mediating CASTOR1 dimerization are indicated by green arrows and purple arrows, respectively. (**d**) Mutation of L22R or I202D disrupted dimerization of CASTOR1, as assayed by the SEC-MALS experiment. (**e**) Structure of a protomer of CASTOR1. The NTD and the CTD domains of CASTOR1 are colored in cyan and green, respectively. The bound arginine is shown in red.

By using the single-wavelength anomalous dispersion (SAD) method with a selenomethionine (SeMet) derivative, we determined the crystal structure of full-length human CATSOR1 in complex with arginine to 2.07 Å resolution, with the *R*_work_/*R*_free_ factors being 19.00%/23.83% (Table 1). In the crystal structure, CASTOR1 forms a dimer with the two protomers related by a two-fold rotation axis, and each protomer contains a bound arginine (Figure 1a). Dimerization buries ~935 Å^2^ of surface area for each protomer, and is mainly mediated through juxtaposition of the *α*1 and *α*5 helices of CASTOR1 (Figure 1b and c). Ile28 and Ile202 from both protomers pack together by hydrophobic interactions; and Trp21, Leu22, and Pro64 from both protomers also gather up to form the van der Waals interactions with each other. In addition, Tyr207 of one protomer donates a hydrogen bond to His25 of the other protomer; and there exist salt bridges between Arg36 and Asp203 from different protomers. Consistent with our structural observation, the CASTOR1 protein in solution is a dimer, as assayed by the size exclusion chromatography—multi-angle static light-scattering (SEC-MALS) method (Supplementary Figure S1). In contrast, mutation of L22R or I202D disrupted dimerization of CASTOR1 (Figure 1d).

Classical ACT domains possess a *βαββαβ* topology[18, 19]. Yet, our structure shows that each protomer of CASTOR1 actually contains four, rather than the proposed two [17], ACT domains. ACT1 (residues 1–75) and ACT2 (residues 76–153) assemble into the N-terminal domain (NTD, residues 1–153), while ACT3 (residues 175–260) and ACT4 (residues 261–329) form the C-terminal domain (CTD, residues 175–329). Both NTD and CTD have a *ββαββαββαββα* topology (there are two extra *β*-strands at the C-terminal end of NTD) (Supplementary Figure S2) and highly resemble each other, with the root-mean-square deviation (RMSD) being 1.053 Å for 80 aligned C*α* atoms (Supplementary Figure S3). The NTD and CTD are reminiscent of two halves of a sphere, and the bound arginine molecule is encased inside this sphere (Figure 1e).

### The arginine-binding site of CASTOR1

**Figure 2:**
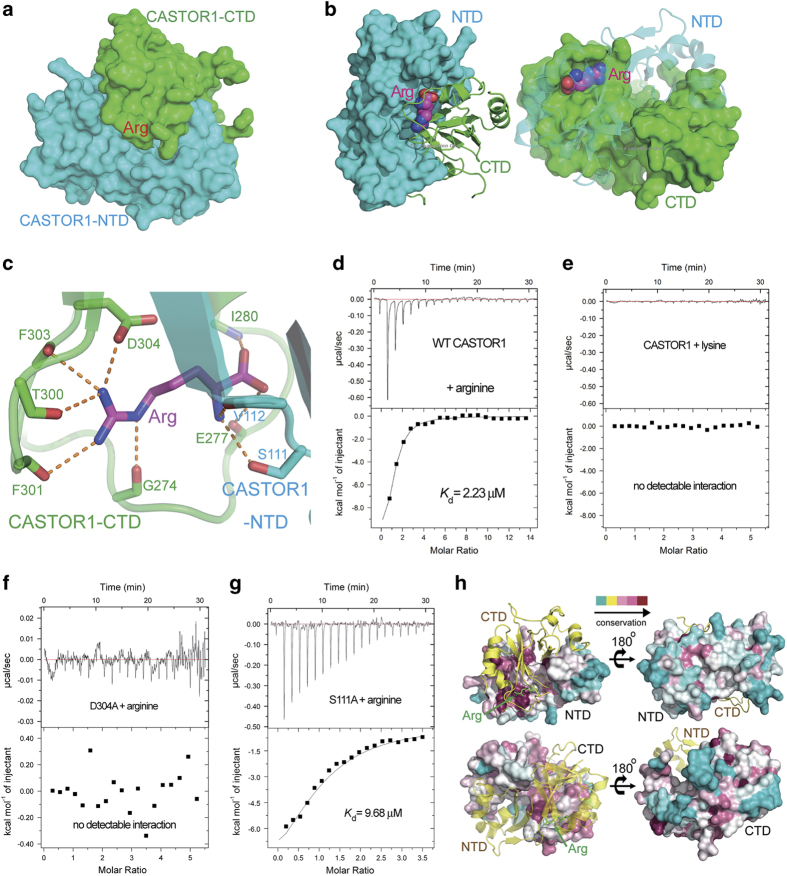
The arginine-binding pocket of CASTOR1. (**a**) The arginine is buried between the NTD and the CTD domains of CASTOR1. (**b**) Both the NTD and the CTD of CASTOR1 employ highly complementary surfaces to recognize the arginine, which is shown in sphere representation. (**c**) The arginine-binding pocket of CASTOR1. Residues from CASTOR1-NTD or CASTOR1-CTD are colored in cyan or green, respectively. The bound arginine is colored in magenta. Hydrogen bonds are represented as orange dashed lines. (**d**) The dissociation constant (*K*_d_) between wild-type (WT) CASTOR1 and arginine was measured by ITC to be 2.23 μM. (**e**) No measurable interaction was detected between CASTOR1 and lysine. (**f**) Point mutation of Asp304 to alanine disrupted the association between CASTOR1 and arginine. (**g**) The S111A mutation diminished the binding affinity of CASTOR1 for arginine, with the *K*_d_ value increasing to 9.68 μM. (**h**) The arginine-binding sites on CASTOR1-NTD and -CTD are highly conserved, whereas the outside surface of CASTOR1 is not conserved.

The arginine molecule is completely buried inside CASTOR1, and is located at the interface between CASTOR1-NTD and -CTD (Figure 2a and Supplementary Figure S4). Both NTD and CTD employ a surface pocket highly complementary to the shape of arginine (Figure 2b), and residues from both domains contribute to its specific recognition. The side-chain carboxyl group of Asp304 and the main-chain carbonyl groups of Gly274, Thr300, Phe301, and Phe303, all from CASTOR1-CTD, form charge-stabilized hydrogen bonds with the guanidinium group of arginine (Figure 2c). In addition, the side-chain hydroxyl group of Ser111 and the main-chain carbonyl group of Val112, both from CASTOR1-NTD, accept hydrogen bonds from the main-chain amino group of arginine (Figure 2c). Besides, the main-chain amino group of Ile280 and the main-chain carbonyl group of Glu277, both from CASTOR1-CTD, make hydrogen bonds with the main-chain carboxyl group of arginine (Figure 2c).

To verify our structural observations, we performed point mutations on key residues in the arginine-binding pocket of CASTOR1, and carried out the isothermal titration calorimetry (ITC) assay to measure their binding affinities for arginine. In contrast to wild-type (WT) CASTOR1 whose dissociation constant (*K*_d_) for arginine was measured to be 2.23 μM (Figure 2d and Table 2), both double mutation of S111A/D304A (Supplementary Figure S5 and Table 2) and single mutation of D304A completely abrogated arginine binding (Figure 2f and Table 2), and single mutation of S111A resulted in an increase of *K*_d_ to 9.68 μM (Figure 2g and Table 2). Interestingly, WT CASTOR1 exhibited no detectable association with lysine (Figure 2e and Table 2), which among the twenty natural l-amino acids is the most similar one to arginine in terms of charge and size. Leucine, another amino acid that has an important role in the regulation of mTORC1 pathway[28, 29], also has no measurable interaction with CASTOR1 (Supplementary Figure S6 and Table 2). This indicates that the sensing of arginine by CASTOR1 is highly specific and selective. The arginine-binding sites on both CASTOR1-NTD and -CTD are highly conserved, whereas the outside surface of CASTOR1 displayed no similar extent of conservation (Figure 2h).

### Arginine tethers the NTD and the CTD domains of CASTOR1 together

**Figure 3:**
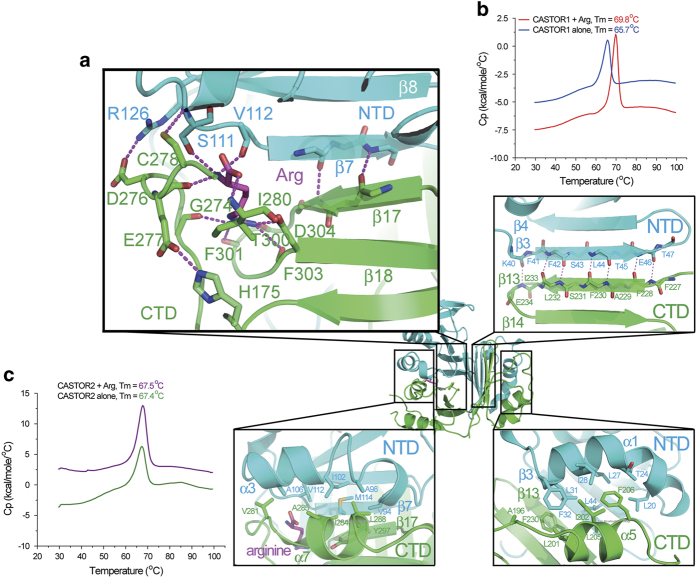
Arginine tethers CASTOR1-NTD and CASTOR1-CTD tightly together. (**a**) Arginine interacts with both the NTD and the CTD of CASTOR1, thus tethering the NTD and the CTD domains tightly together. The other three interfaces between CASTOR1-NTD and -CTD are also shown. (**b**) The DSC assay demonstrated that arginine promoted tighter folding of CASTOR1, as shown by the increase of its melting temperature (*T*_m_). (**c**) The DSC assay revealed that the melting temperature of CASTOR2 was not affected by the addition of arginine.

As described above, the NTD and the CTD domains of CASTOR1 resemble two halves of a sphere. Their association results in closure of the sphere and encasement of the bound arginine. The NTD and the CTD interact through four layers of interfaces: the one close to NTD-*β*7 and CTD-*β*17, which also involves the contribution of the bound arginine (Figure 3a, top left), the *β*-sheet formation between NTD-*β*3 and CTD-*β*13 (Figure 3a, top right), the pairing between two helices NTD-*α*3 and CTD-*α*7 (Figure 3a, bottom left), and the juxtaposition of NTD-*α*1 and CTD-*α*5 helices (Figure 3a, bottom right).

Compared with the second interface where the coupling between NTD-*β*3 and CTD-*β*13 results in the formation of seven hydrogen bonds, there are only two hydrogen bonds contributed by the pairing of NTD-*β*7 and CTD-*β*17 at the first interface, which is the most likely entrance for the arginine to squeeze into the CASTOR1 molecule. The admission of arginine further tethers the NTD and the CTD domains of CASTOR1 together by binding to residues from both the NTD (such as Ser111) and the CTD (such as Asp304). In addition, residues from the loop between *β*16 and *α*7, which belongs to the CTD, make further contribution to the interaction between CASTOR1-NTD and -CTD. Asp276 forms electrostatic interaction with Arg126 from the NTD, while Glu277 makes a charge-stabilized hydrogen bond with His175, which is close to the NTD. Besides, there is another hydrogen bond between Cys278 and the main-chain of Ser111 from the NTD (Figure 3a). Therefore, the first interface is the most probable site for arginine to enter CASTOR1, and, when bound, it strengthens the association between the NTD and the CTD which is further stabilized by the loop between *β*16 and *α*7.

In agreement with our structural observation, the melting temperature (*T*_m_) of CASTOR1 in the presence and in the absence of arginine was measured to be 69.8 °C and 65.7 °C, respectively, by the differential scanning calorimetry (DSC) assay (Figure 3b). Therefore, the enclosure of arginine causes the CASTOR1 protein to fold more tightly, and leads to an almost 4 °C of increment in its *T*_m_. In contrast, CASTOR2, which did not have detectable binding affinity for arginine (Supplementary Figure S7), did not exhibit much change in *T*_m_ in the presence or absence of arginine (Figure 3c).

### CASTOR1 employs a surface patch on its NTD to interact with the GATOR2 subunit Mios

As noted from its sequence homology [17], the structure of CASTOR1 bears similarity to those of other ACT domain-containing proteins, especially the ACT domains of *Escherichia coli* aspartate kinase (PDB code: 2J0X) [30] and cyanobacteria aspartate kinase (PDB code: 3L76) [31]. Similar to CASTOR1, these prokaryotic aspartate kinases also contain bound amino acids. There are two bound lysine molecules for the ACT domain of *E. coli* aspartate kinase (Supplementary Figure S8a), while two lysines and two threonines are associated with the ACT domain of the cyanobacteria aspartate kinase (Supplementary Figure S8b). Their secondary structure organizations and binding sites for amino acids are all similar to those of CASTOR1 (Supplementary Figure S8c).

**Figure 4:**
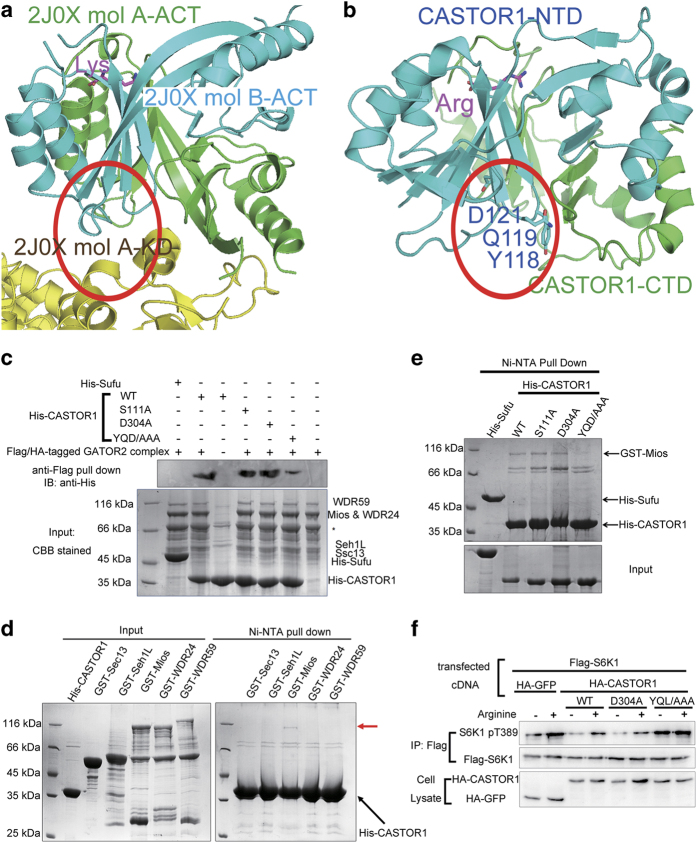
A surface patch on CASTOR1-NTD on the opposite side of the arginine-binding site mediates its association with GATOR2. (**a**) The ACT domain of *E. coli* aspartate kinase (PDB code: 2J0X) employs a surface patch on the opposite side of its lysine-binding site to associate with its KD. In this structure, *E. coli* aspartate kinase exists as a dimer. The two protomers are denoted as mol A and mol B, respectively. (**b**) A surface patch on CASTOR1-NTD at a similar position as the *E. coli* aspartate kinase ACT domain’s binding site for its KD domain was identified to mediate its association with GATOR2. (**c**) Mutation of Y118A/Q119A/D121A on CASTOR1 decreased its interaction with the purified GATOR2 complex. His-tagged Suppressor of Fused (Sufu) protein served as a control. (**d**) Out of the five subunits of GATOR2, only Mios exhibited direct physical interaction with CASTOR1, as assayed by the Ni^2+^-NTA pull-down experiment using purified proteins. (**e**) Mutation of Y118A/Q119A/D121A on CASTOR1 almost abrogated its interaction with the purified Mios protein, a component of the GATOR2 complex. His-tagged Sufu served as a control. (**f**) Mutation of Y118A/Q119A/D121A abolished CASTOR1’s ability to inhibit GATOR2, and the downstream mTORC1 kinase phosphorylated S6K1 even in the absence of arginine.

In the structure of *E. coli* aspartate kinase, there is an N-terminal kinase domain (KD) and a C-terminal ACT domain. The ACT domain’s interaction interface for the KD is on the opposite side to its lysine-recognition pocket (Figure 4a). Hence, the lysine serves as a ligand for the ACT domain which allosterically regulates its association with the KD. Through comparison, we hypothesized that the surface patch on CASTOR1-NTD involving Tyr118, Gln119, and Asp121, opposite to where its ligand arginine binds, might be the interface for association with its downstream effector, the GATOR2 complex (Figure 4b). Indeed, triple mutation of Y118A/Q119A/D121A on CASTOR1 pulled down much less purified GATOR2 protein complex (Figure 4c). Interestingly, mutation of D304A or S111A, which abrogated or decreased arginine binding (Figure 2f and g), enhanced the amount of GATOR2 complex pulled down by CASTOR1 (Figure 4c).

Mios, one out of the five subunits of GATOR2, was reported to participate in the association with CASTOR1 [17]. We employed the Ni^2+^-NTA pull-down assay to examine the interaction between each of the five subunits of GATOR2 and CASTOR1 using purified proteins, and found that among the five subunits of GATOR2, only Mios exhibited considerable direct physical interaction with CASTOR1 (Figure 4d). In addition, by using the Ni^2+^-NTA pull-down assay with purified CASTOR1 and Mios proteins, we found that the Y118A/Q119A/D121A mutation, as compared with the WT control, caused substantial dissociation of Mios from CASTOR1 (Figure 4e). This is also the first experimental evidence, to our knowledge, that there exists direct interaction between CASTOR1 and Mios proteins. Furthermore, the Y118A/Q119A/D121A mutation disabled CASTOR1’s ability to inhibit the downstream mTORC1 signaling, such as S6K1 phosphorylation, even in the absence of arginine (Figure 4f).

### Normal mode analysis suggests an ‘open’-to-‘closed’ conformational switch for CASTOR1

**Figure 5:**
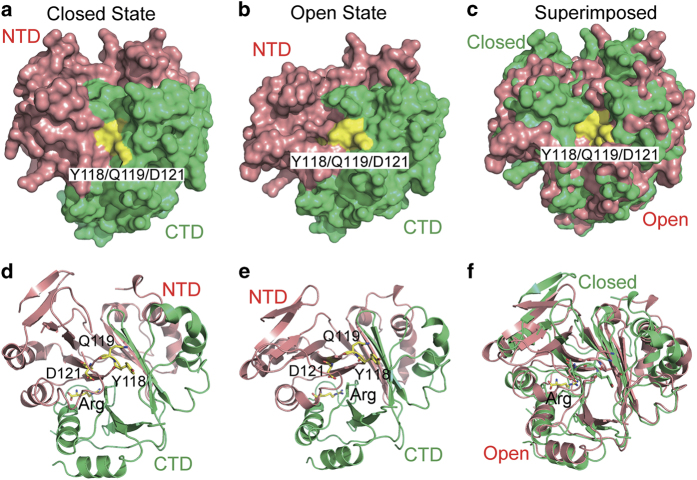
Normal mode analysis suggests that there exists an ‘open’-to-‘closed’ conformational switch for CASTOR1. (**a**, **d**) In the ‘closed’ state of CASTOR1 which resembles more closely to the crystal structure, the NTD and the CTD domains are more compressed against each other, and the GATOR2-binding residues are mostly buried. (**b**, **e**) In the ‘open’ state of CASTOR1, the NTD and the CTD domains are more separated, and the GATOR2-binding residues are exposed. (**c**, **f**) Superimposition of the 'closed’ and the ‘open’ states of CASTOR1. (**a**–**c**) Surface representations. (**d**–**f**) Cartoon representations.

To investigate potential conformational rearrangement of CASTOR1, we subjected our structure to the NM analysis [32]. Interestingly, the NM analysis revealed a breathing motion between the NTD and the CTD domains of CASTOR1. In the ‘closed and compact’ state which resembles more closely to the crystal structure, the NTD and the CTD domains of CASTOR1 are compressed against each other tightly. As a result, the GATOR2-binding residues Y118/Q119/D121 are mostly buried and not available for contact with GATOR2 (Figure 5a and d). In contrast, in the ‘open and relaxed’ state, the NTD and the CTD domains of CASTOR1 are much more separated and the GATOR2-binding surface patch is exposed, ready for interaction with GATOR2 (Figure 5b and e). Presumably, this conformational switch (Figure 5c and f and Supplementary Video S1) of CASTOR1 is correlated with arginine-binding. In the absence of arginine, CASTOR1 exists in the ‘open’ state. Its GATOR2-binding surface patch is exposed and associates with GATOR2. On the other hand, when arginine is present, the binding of arginine tethers the NTD and the CTD domains of CASTOR1 tightly together. Thus a conformational rearrangement of CASTOR1 from the ‘open’ state to the ‘closed’ state is induced, and the GATOR2-binding surface patch of CASTOR1 is concealed. Hence GATOR2 is detached from CASTOR1 and is free to activate the downstream mTORC1 signaling.

## Discussion

Arginine has a critical role in the regulation of many aspects of mammalian physiology, such as insulin secretion, intestinal cell migration, immune responses, and so on [33–35]. It is an interesting question of how cells sense the presence of arginine and transmit its signal. Our structure of the CASTOR1-arginine complex reveals that a nicely tailored pocket on CASTOR1 is employed to recognize arginine, but not other common amino acids such as lysine and leucine (Figure 2). Furthermore, our NM analysis reveals that there is an intrinsic flexibility between the two domains of CASTOR1, and the relative movement between its two domains underlies its association/dissociation with its downstream effector GATOR2 (Figure 5), which is most likely regulated by arginine binding. This kind of intrinsic inter-domain flexibility and conformational switching regulated by a binding partner is reminiscent of other key mediators in important signal transduction pathways [36]. It will be interesting to elucidate how exactly arginine binding induces the inter-domain dynamic movement of CASTOR1.

Human CASTOR2 is a close homolog of CASTOR1, but it does not recognize arginine (Table 2) and it is constitutively associated with GATOR2. Our DSC assay suggests that it folds tighter than CASTOR1 (Supplementary Figure S9). Comparison of primary sequences of CASTOR1 orthologues and CASTOR2 orthologues suggested that residues at positions of 108–110 and 302 (CASTOR1 residue numbers; H108/H109/V110 and N302 for CASTOR1, while Q110/N111/I112 and K303 for CASTOR2) might be responsible for their different arginine-binding abilities (Supplementary Figure S10). Occurrence of amimo acids different from CASTOR1 at these positions of CASTOR2 might underlie its incapability to arginine, which is consistent with the findings of other groups [37, 38]. Regrettably, we were not able to obtain diffracting crystals of CASTOR2 despite considerable efforts. It remains an important question of how the structure of CASTOR2 differs from CASTOR1. This structural information might provide an answer to why it does not interact with arginine but associates with GATOR2 constitutively.

The GATOR2 complex, which is the downstream effector of CASTOR1, consists of five subunits, Mios, WDR59, WDR24, Seh1L, and Sec13 [12]. In a previous report, WDR24, Mios, and Seh1L have been implicated in CASTOR1 binding [17]. However, it is still unknown which subunit(s) provides direct physical contact with CASTOR1. Using purified proteins, we found that among the five subunits of GATOR2, only Mios directly interacts with CASTOR1. It would be interesting to further examine the structural mechanism of how Mios associates with CASTOR1.

In the mean time when this manuscript was being prepared, reports on the crystal structure of CASTOR1 in complex with arginine were published by the Sabatini and co-workers [37], as well as by the Ding and co-workers [38]. Their reports obtained similar structural observations as ours, both on the overall structure and the arginine-binding pocket. In Xia *et al.* [38], the dissociation constant *K*_d_ between CASTOR1 and arginine was measured by ITC to be 5.5 μM, which is similar to our result. On the other hand, in Chantranupong *et al.* [17], the *K*_d_ value was found to be 34.8 μM, as assayed through measuring the amount of [^3^H] radio-labeled arginine pulled down by immunoprecipitated CASTOR1. This discrepancy might be caused by the difference between *in vivo* *versus* *in vitro* assay methods. Within cells, there might be other arginine-binding proteins other than CASTOR1, which would compete with it for arginine binding and decrease the effective binding affinity between CASTOR1 and arginine. In Saxton *et al.*[37], they also revealed that Y118/Q119/D121 of CASTOR1 were involved in GATOR2-binding and mutation of Y118/Q119/D121 disrupted CASTOR1-binding to GATOR2 and activated downstream S6K1 phosphorylation in the absence of arginine. However, their *in vivo* co-IP approach could not exclude the possibility that some other protein(s) might mediate the interaction between CASTOR1 and Mios. Our *in vitro* pull-down result using purified proteins is complementary to their finding, and provides direct evidence for physical interaction between residues Y118/Q119/D121 of CASTOR1 and Mios, but not the other four subunits of GATOR2.

**Figure 6:**
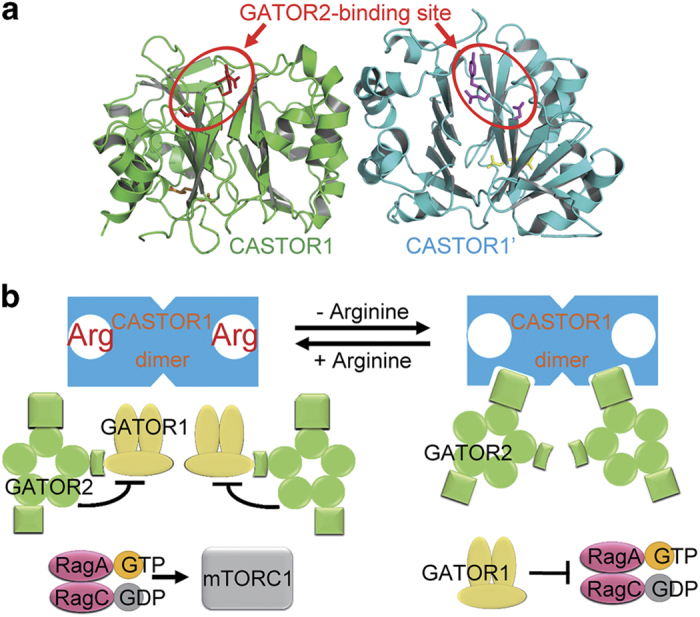
Dimerization of CASTOR1 might have a critical role in relieving the inhibition of GATOR1 by GATOR2. (**a**) In the structure of CASTOR1 dimer, the GATOR2-binding sites (residues Y118/Q119/D121, marked by red circles) on the two CASTOR1 protomers are located on the same side and face the same direction. (**b**) A schematic model showing that the dimerization of CASTOR1 leads to juxtaposition of two GATOR2 complexes on the relatively small CASTOR1 dimer, which creates spatial hindrance for their bound GATOR1 complexes. GATOR1 is thus dissociated from GATOR2 and relieved from its inhibition.

Both CASTOR1 and CASTOR2 function as dimers to relieve the inhibition of GATOR1 by GATOR2 [17], so that GATOR1 can prevent the RagA/RagB−RagC/RagD complex from activating mTORC1. Interestingly, it was reported that dimerization-deficient CASTOR1 mutant displayed weaker interaction with GATOR2 and failed to inhibit downstream mTORC1 signaling [37]. Examination of the structure of CASTOR1 dimer revealed that the GATOR2-binding sites (that is, residues Y118/Q119/D121) of both CASTOR1 protomers reside on the same side and face the same direction (Figure 6a). This inspired us to propose a model of how dimerization of CASTOR1 (and maybe CASTOR2 as well) relieves the inhibition of GATOR1 by GATOR2 (Figure 6b). In the presence of arginine, GATOR2 dissociates from CASTOR1, and binds to GATOR1 to inhibit its GAP activity towards RagA/RagB. GTP-bound RagA/RagB in complex with GDP-bound RagC/RagD activates mTORC1 (Figure 6b, left). On the other hand, when arginine is absent, the GATOR2-binding site on CASTOR1 is exposed, and each of the CASTOR1 protomer is bound by a GATOR2 complex. Juxtaposition of two gigantic GATOR2 complexes (~378 kDa for a GATOR2 complex) on the relatively small CASTOR1 dimer (~36 kDa for a CASTOR1 momomer) creates severe spatial hindrance for the two GATOR1 complexes associated with GATOR2, which are also huge (~285 kDa for a GATOR1 complex). Therefore, steric clash would force GATOR1 to dissociate from GATOR2, hence stimulating the hydrolysis of GTP bound with RagA/RagB and inhibiting the downstream mTORC1 activity (Figure 6b, right). Interestingly, to our knowledge, similar dimeric assembly as that of CASTOR1 is not found in bacterial proteins possessing ACT domains such as aspartate kinases. We hypothesize that the dimeric assembly of CASTOR proteins specifically evolves to engage the GATOR2/GATOR1 system, which is present in metazoa but not in prokaryotes.

## Methods

### Protein expression and purification

Full-length human CASTOR1 and CASTOR2 were subcloned into the pET28a vector, with an N-terminal 6×His-tag. The cDNA encoding full-length human Mios was subcloned into a pGEX4T-1 vector, with an N-terminal GST tag. These vectors were transformed into *E. coli* BL21(DE3)-competent cells. Transformed cells were cultured at 37  °C until the optical density (OD) at 600 nm was 0.8, and protein expression was induced with 0.2 mM IPTG for 14–18 h at 16 °C. After harvesting and resuspension, cells were lysed with a cell homogenizer (JNBIO, Guangzhou, China). The lysate was cleared by centrifugation at 14 000 r.p.m. for 30 min and the supernatants were subjected to Ni^2+^-NTA or GST affinity chromatography (GE healthcare, Little Chalfont, UK). The eluted protein was loaded onto a Superdex 200 gel filtration column (GE Healthcare) in 15 mM Tris-HCl, pH 8.0, 150 mM NaCl, and 3 mM dithiothreitol (DTT). Peak fractions were combined, supplemented with 2 mM arginine, and incubated for 30 min before being concentrated to 10 mg ml^−1^ for crystallization. Constructs of CASTOR1 mutants were derived by the whole plasmid PCR/DpnI digestion method, and were verified by sequencing. All the mutant CASTOR1 proteins were purified using the same procedure as for the WT proteins. Selenomethionine (SeMet)-derivatized CASTOR1 was expressed using the methionine-autotrophic *E. coli* B834(DE3) cells cultured in M9 media and purified as the native protein, except that 5 mM DTT was used in the gel filtration buffer.

### Crystallization, data collection, and structure determination

Native and SeMet-substituted human CASTOR1 protein crystals were grown at 14 °C by the hanging-drop vapor-diffusion method, with 1 μl of protein mixed with an equal volume of reservoir solution containing 18% PEG 3 350 and 0.2 M magnesium acetate. Crystals were cryoprotected in the crystallization buffer supplemented with 25% glycerol. One set of single-wavelength anomalous dispersion (SAD) data of SeMet-CASTOR1 at 1.96 Å resolution was collected at the beamline BL18U1, and one set of data of native CASTOR1 at 2.07 Å resolution was collected at the beamline BL19U1 of National Center for Protein Sciences Shanghai at 100 K. Data reduction was performed with the HKL3000 software [39]. The SAD phases for the SeMet-CASTOR1 were determined using the Autosol module of PHENIX [40], and the phases for the native CASTOR1 were determined using the Phaser [41] program in the CCP4 package [42] After automatic model-building using the AutoBuild module of PHENIX [40] and manual model-building in Coot [43], refinement was performed by the REFMAC5 program [44] in the CCP4 package [42]. The crystals belonged to the space group *P*2_1_, and contained two molecules of CASTOR1 in one asymmetric unit. The final refined model has *R*_work_/*R*_free_ factors of 19.00%/23.83%. The quality of the structural model was checked with the CCP4 program PROCHECK [42], which shows a good stereochemistry according to the Ramachandran plot.

### Isothermal titration calorimetry (ITC) and differential scanning calorimetry (DSC) assay

ITC experiments were performed with an ITC200 system (MicroCal, Worcestershire, UK) at 25 °C as described before [45]. Both ITC and DSC reaction buffers contained 25 mM HEPES-Na, pH 7.5, and 150 mM NaCl. All samples and buffer were centrifuged and degassed before experiments. To determine the *T*_m_ values of CASTOR1 and CASTOR2 protein, VP-DSC (MicroCal) was used to measure the excess heat capacity of protein unfolding. For DSC analysis, protein samples at 1–2 mg/ml were loaded into the sample cell and the reaction buffer was taken as the reference. The heat capacity change was measured from 30  to 100 °C with a scan rate of 90 °C/h and system pressure ≥35 p.s.i. Buffer baseline scans were established before analysis of each protein sample. Data were analyzed using the Origin 7.0 software. Sample scans were buffer-subtracted and concentration-normalized.

### Cell culture, transfection, and plasmids

Expi293F cells (Thermo Fisher Scientific, Waltham, MA, USA) were cultured in the Expi293 Expression medium (Thermo Fisher Scientific) on a shaker with 5% CO_2_ at 37 °C. Cell transfection was performed using ExpiFectamine293 Transfection Kit (Thermo Fisher Scientific) as described in the manual. The pRK5 plasmids expressing Flag-WDR24, Flag-Mios, HA-WDR59, HA-Sec13, and HA-Seh1L were obtained from Addgene (Cambridge, MA, USA). The cDNAs encoding human CASTOR1 and human CASTOR2 were chemically synthesized (Union-Biotech, Shanghai, China).

### *In vitro* CASTOR1-GATOR2 dissociation assay

Expi293F cells were harvested 48 h after co-transfection of the five subunits of GATOR2, and cells were undergone three cycles of freeze-and-thaw in the lysis buffer (50 mM Tris-HCl, pH 8.0, 150 mM NaCl, supplemented with the protease inhibitor cocktail). After centrifugation at 14 000 r.p.m. for 50 min at 4 °C, the supernatants were subjected to affinity chromatography using the anti-FLAG M2 resin (Sigma, St Louis, MO, USA). After incubation at 4 °C for 1 h, the resin was washed thoroughly with the lysis buffer, and 200 μg ml^−1^ FLAG peptide (with a sequence of DYKDDDDK) was used to elute bound proteins. After SDS-PAGE analysis, the eluted protein was subjected to the Superdex 200 gel filtration chromatography to remove the FLAG peptide.

The purified GATOR2 protein complex was mixed with His-tagged WT or mutant CASTOR1 protein with a molar ratio of 1:3. After 30 min of incubation at 4 °C, the mixture was loaded onto an anti-FLAG affinity column. Bound proteins were eluted with buffer containing the FLAG peptide, and anti-His immunoblot was carried out to detect the bound CASTOR1 pulled down by the GATOR2 complex. His-tagged Suppressor of Fused (Sufu) protein was used as a control.

Binding assays between purified WT or mutant CASTOR1 proteins and WT GST-Mios were performed by the Ni^2+^-NTA column pull-down assay. The GST-Mios protein was added to His-CASTOR1 protein pre-immobilized on the Ni^2+^-NTA affinity column (GE Healthcare) at 4 °C, washed with the Ni^2+^-NTA column buffer (25 mM Tris, pH 8.0, 300 mM NaCl, and 20 mM imidazole) and eluted with a buffer containing 25 mM Tris, pH 8.0, 300 mM NaCl, and 500 mM imidazole. Eluted protein fractions were analyzed by SDS-PAGE and Coomassie Blue staining.

### Normal mode analysis

The NM analysis was performed as described before [36, 46]. Briefly, the structural coordinates of CASTOR1 were submitted for the NM analysis using the Elastic Network Model server (http://www.igs.cnrsmrs.fr/elnemo/) [32], which is a fast and simple tool to compute the major (that is, low-frequency) NMs of a protein. The first vibrational mode (that is, the seventh NM, the first six modes being translational and rotational motions of the system as a whole) generated by the server, which showed the clearest inter-domain movement between NTD and CTD and was correlated with the burying and exposing of the GATOR2-binding residues, was selected for further analysis.

### Size exclusion chromatography—multi-angle static light-scattering (SEC-MALS) assay

SEC-MALS experiments were carried out at 25 °C with a DAWN HELEOS II 18-angle light-scattering instrument (Wyatt Technology, Santa Barbara, CA, USA) and a size exclusion chromatography WTC-015S5 column (Wyatt Technology) coupled to an Agilent 1 100 HPLC system (Agilent, Santa Clara, CA, USA). Approximately 400 μg protein was injected per run, in a buffer containing 15 mM Tris-HCl, pH 8.0, 150 mM NaCl, and 2 mM DTT. The molecular weight of each sample was calculated by the ASTRA 6.1 software (Wyatt Technology).

### S6K1 phosphorylation assay

About 0.8 million HEK-293 T cells were plated in a 6-cm cell culture dish. After 24 h, 5 μg HA-CASTOR1 or HA-GFP and Flag-S6K1 constructs were co-transfected. 36 h later, cells were starved of arginine for 45 min with arginine-free DMEM medium or starved for 45 min and restimulated with 1 mM arginine in cell media for 15 min. Cells were rinsed with chilled PBS and lysed with Triton lysis buffer (1% Triton X-100, 25 mM Tris-HCl pH 7.4, 100 mM NaCl, 10 mM KCl, and 2.5 mM MgCl_2_) supplemented with EDTA-free protease inhibitor (Roche, Basel, Switzerland). The soluble lysate fractions were isolated by centrifugation at 14 000 r.p.m. for 15 min at 4 °C. The FLAG-M2 resin (Sigma) was added and incubated with the cell lysate for 4–6 h at 4 °C. The beads were washed five times with the lysis buffer and boiled with the sample buffer for 5 min. Samples were resolved by 12% SDS-PAGE and analyzed by immunoblot. Phospho-S6K1 (Thr389) antibody (Cell Signaling Technology, Beverly, MA, USA) was employed to detect phospho-S6K1.

### Molecular graphics

All protein structure figures were generated by the PyMOL program [47]. Sequence conservation of CASTOR1 mapped onto the surface of its crystal structure was generated by the ConSurf server (http://consurf.tau.ac.il) [48].

### Accession codes

The atomic coordinates and structure factors of the complex between full-length human CASTOR1 and arginine have been deposited in the Protein Data Bank with the accession number 5GV2.

## Figures and Tables

**Table 1:** Data collection and refinement statistics

	*SeMet-CASTOR1−arginine*	*CASTOR1−arginine complex*
*Data collection*		
Space group	C2	P21
*Cell dimensions*		
a, b, c (Å)	95.86, 77.50, 48.09	47.55, 76.83, 95.22
*α*,* β*,* γ* (°)	90, 94.02, 90	90, 96.21, 90
Resolution (Å)	50–1.96 (2.03–1.96)	50–2.07 (2.14–2.07)
*R*_merge_ (%)	8.0 (>100.0)	10.0 (99.4)
I/*σ*I	11.3 (1.0)	11.5 (1.2)
CC1/2	0.524	0.583
Completeness (%)	99.8 (99.4)	99.7 (99.3)
Redundancy	3.3 (3.1)	3.3 (3.0)
		
*Refinement*		
Resolution (Å)		50–2.07
No. of reflections		31 497
*R*_work_/*R*_free_		19.00%/23.83%
No. of atoms		
Protein and ligand		4 805
Ion		1
Solvent		149
B-factors (Å^2^)		
Overall		32.12
Protein and ligand		32.11
Ion		10.64
Solvent		32.53
RMSD bond length (Å)		0.0167
RMSD bond angles (°)		1.8714
Ramachandran plot		
Favored (%)		92.6
Additional allowed (%)		7.0
Generously allowed (%)		0.2
Disallowed (%)		0.2

Abbreviations: ASU, asymmetric unit; r.m.s.d., root-mean-square deviations from ideal geometry.

*R*_merge_=Σ_h_Σ_i_ |*I*_h,i_ – *I*_h_|/Σ_h_Σ_i_ *I*_h,i_ for the intensity (*I*) of observation i of reflection h. *R* factor=Σ||*F*_obs_| - |*F*_calc_||/Σ|*F*_obs_|, where *F*_obs_ and *F*_calc_ are the observed and calculated structure factors, respectively. *R*_free_=*R* factor calculated using 5% of the reflection data chosen randomly and omitted from the start of refinement. Data for the highest-resolution shell are shown in parentheses.

**Table 2:** Dissociation constants (*K*_d_) for the interaction between various CASTOR1/CASTOR2 constructs and various amino acids as measured by the isothermal titration calorimetry assay

*CASTOR1/CASTOR2*	*Amino acid*	*Molar ratio*	*K*_*d*_ *(μM)*	*ΔH (kcal mol^−1^)*	*TΔS (kcal mol^−1^)*
WT CASTOR1	Arginine	1.01±0.10	2.23±0.20	−13.4±1.7	−6.05
WT CASTOR1	Lysine	ND	ND	ND	ND
WT CASTOR1	Leucine	ND	ND	ND	ND
WT CASTOR2	Arginine	ND	ND	ND	ND
CASTOR1-S111A	Arginine	0.95±0.09	9.68±3.20	−1.81±0.24	5.19
CASTOR1-D304A	Arginine	ND	ND	ND	ND
S111A/D304A	Arginine	ND	ND	ND	ND

Abbreviation: ND, no detectable interaction was observed.

±values, errors of curve fitting.
