# Supplementary material for: Structural mechanism for the arginine sensing and regulation of CASTOR1 in the mTORC1 signaling pathway
Source: Cell Discov. 2016 Dec 27;2:16051–. doi: 10.1038/celldisc.2016.51 (PMC5187391; doi:10.1038/celldisc.2016.51)
Supplement: Supplementary Information [file celldisc201651-s1.pdf]

Supplementary Information

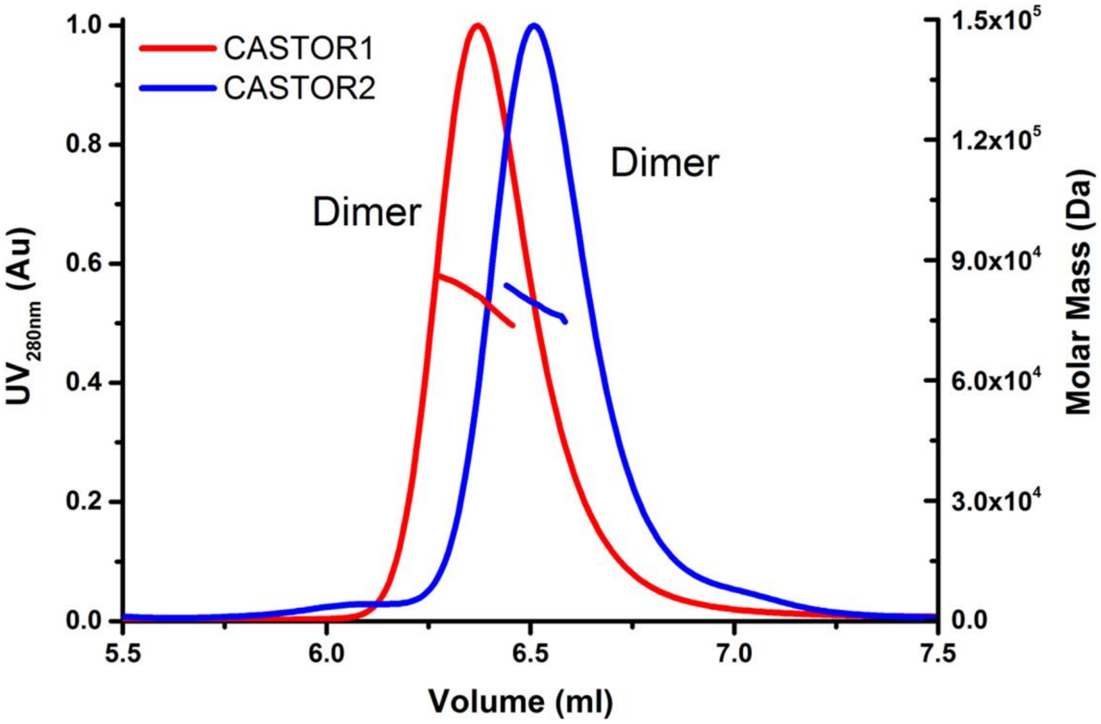

Supplementary Figure S1 Wild type (WT) CASTOR1 and CASTOR2 proteins in solution behaved as dimers, as assayed by the size exclusion chromatography – multi-angle static light scattering (SEC-MALS) method.

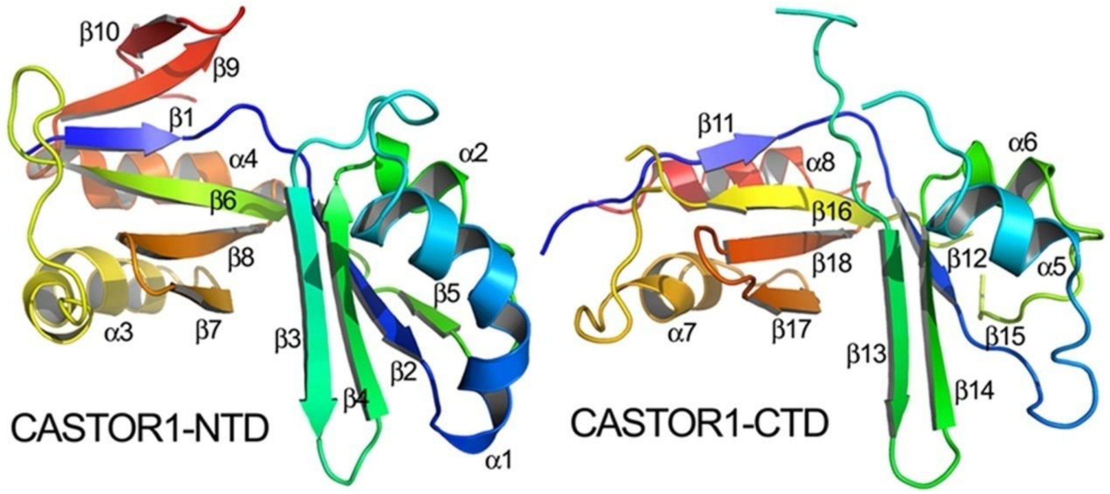

Supplementary Figure S2 Secondary structures of human CASTOR1. Left: secondary structures of the N-terminal domain (NTD) of CASTOR1. Right: secondary structures of the C-terminal domain (CTD) of CASTOR1. The protein is rainbow-colored from blue (N-terminus) to red (C-terminus).

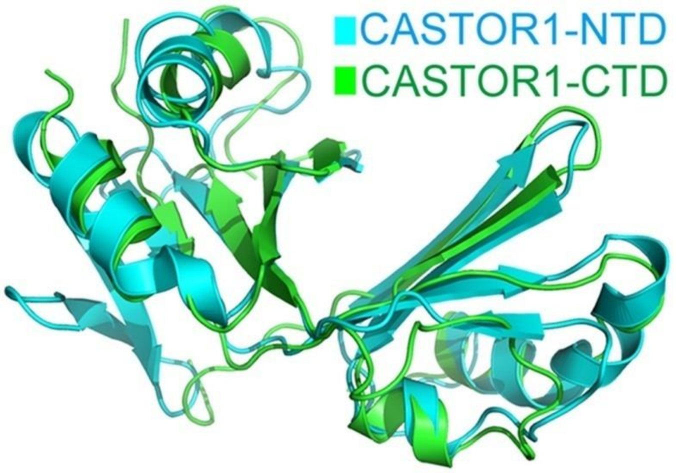

Supplementary Figure S3 Superimposition of the NTD and the CTD domains of CASTOR1. The NTD and the CTD domains of CASTOR1 are colored in cyan and green, respectively.

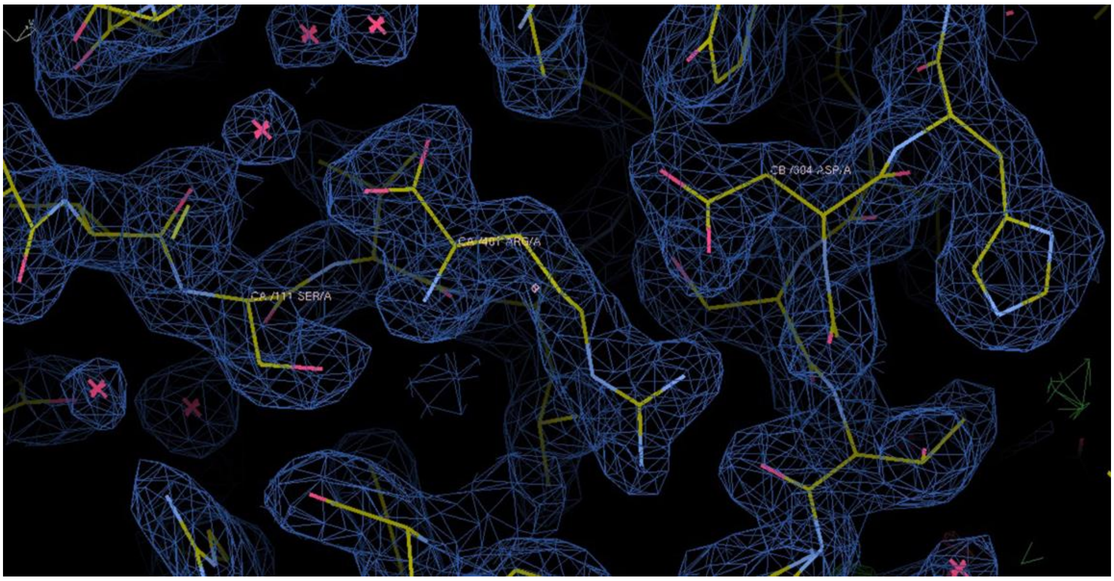

Supplementary Figure S4 The 2*F*o–*F*c electron density map at the CASTOR1-arginine interface. The map is contoured at the 1σ contour level (blue). In the structure model, carbon, oxygen, and nitrogen atoms are colored in yellow, red, and cyan, respectively. Water molecules are denoted as red crosses.

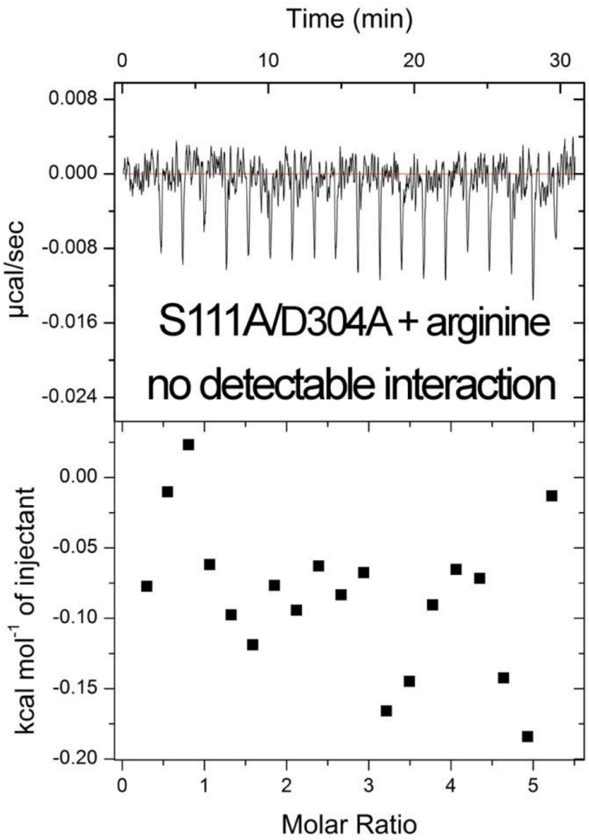

Supplementary Figure S5 No detectable interaction was measured between the S111A/D304A double mutant of CASTOR1 and arginine.

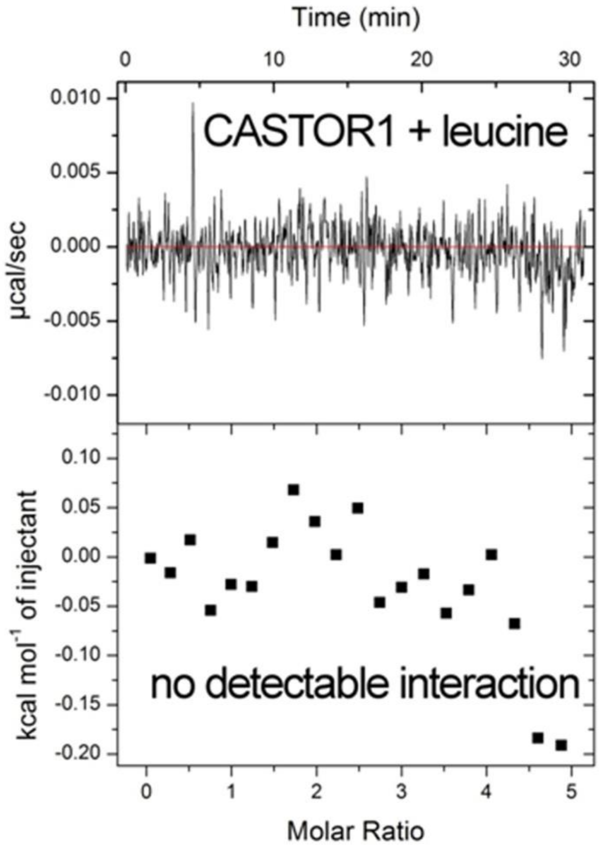

Supplementary Figure S6 No detectable interaction was measured between CASTOR1 and leucine.

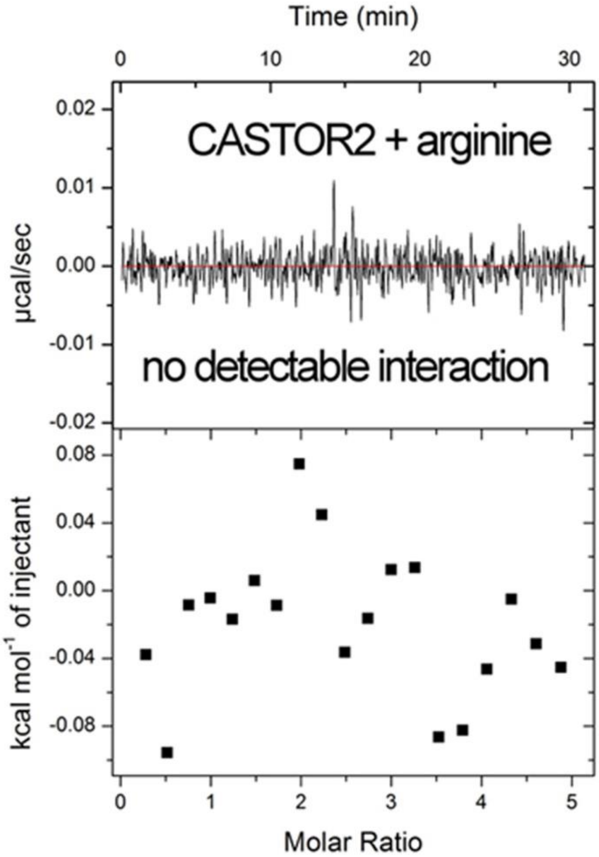

Supplementary Figure S7 No detectable interaction was measured between human CASTOR2 and arginine.

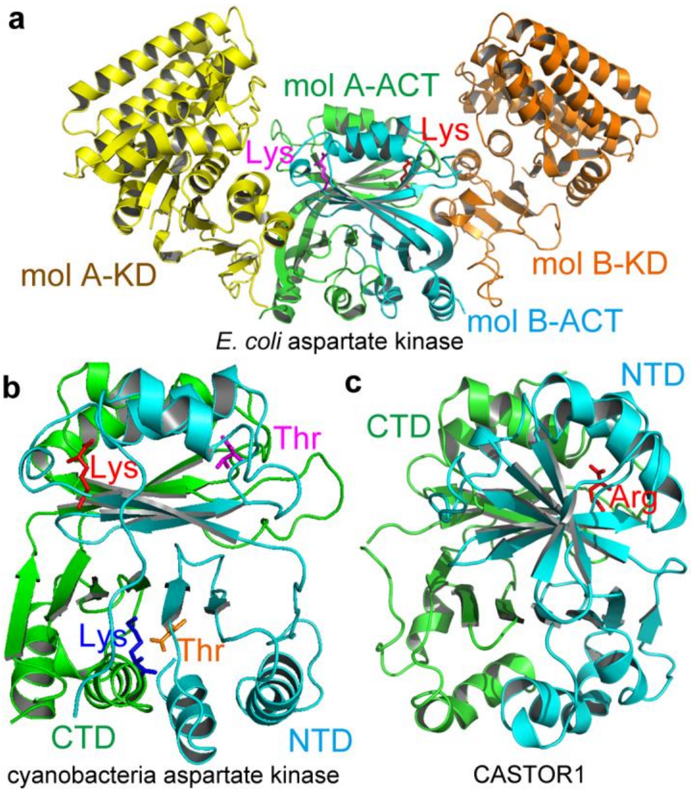

Supplementary Figure S8 CASTOR1 displays structural similarity with *E. coli* and cyanobacteria aspartate kinases. (**a**) The ACT domain of *E. coli* aspartate kinase (PDB code: 2J0X) displays a similar structure as that of CASTOR1. The kinase domains and ACT domains of its two protomers are colored in yellow/orange and green/cyan, respectively. Its bound lysines are shown as sticks and colored in red/magenta. (**b**) The ACT domain of *Synechocystis* (cyanobacteria) aspartate kinase (PDB code: 3L76) also exhibits a similar fold as that of CASTOR1. Its NTD and CTD domains are colored in cyan and green, respectively. Its bound lysines and threonines are shown as sticks and colored in red/blue and magenta/orange, respectively. (**c**) The structure of CASTOR1 is shown as a comparison.

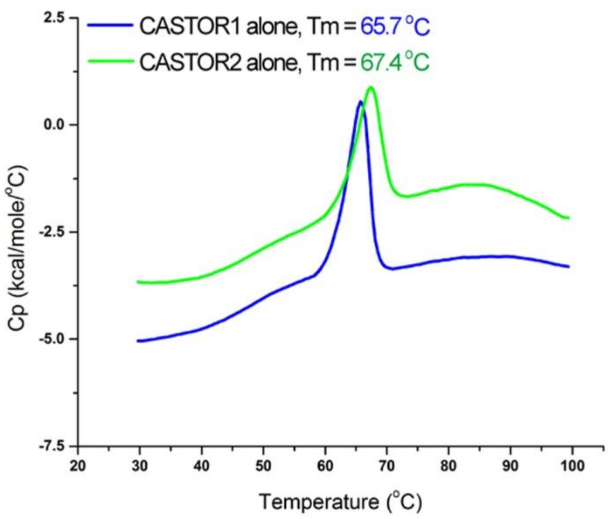

Supplementary Figure S9 CASTOR2 folds tighter than CASTOR1, as reflected by their melting temperatures (T_m). The T_m values of CASTOR2 and CASTOR1 in the absence of arginine were measured to be 67.4℃ and 65.7℃, respectively.

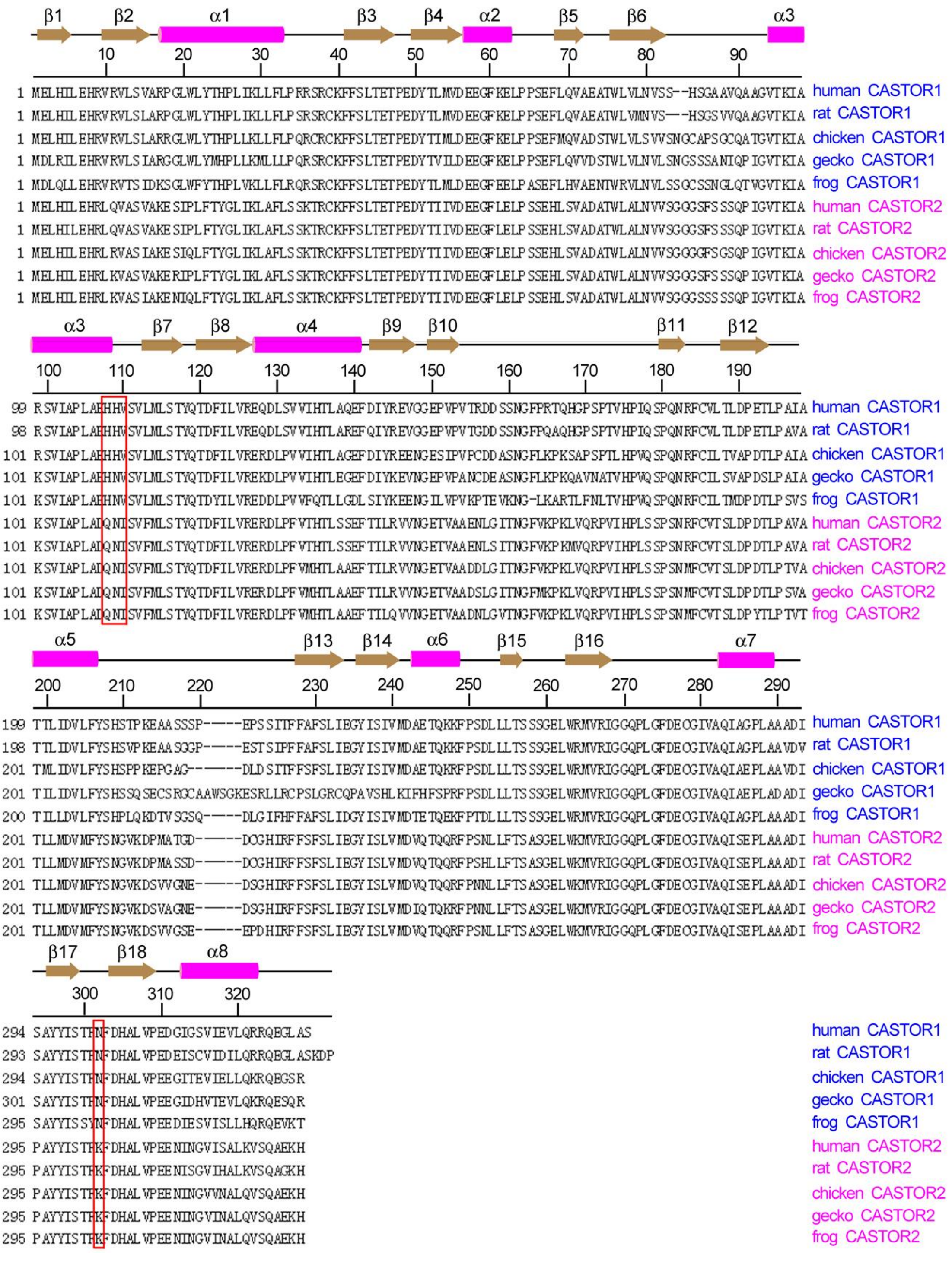

Supplementary Figure S10 Structure-based sequence alignment of human CASTOR1, rat (*Rattus norvegicus*) CASTOR1, chicken (*Gallus gallus*) CASTOR1, gecko (*Gekko japonicus*) CASTOR1, frog (*Xenopus laevis*) CASTOR1, human CASTOR2, rat CASTOR2, gecko CASTOR2, and frog CASTOR2. Candidate residues which might be responsible for the differential

arginine-binding affinities between CASTOR1 and CASTOR2 were marked by red squares.

Supplementary Video S1 Normal mode analysis of CASTOR1. The 1st vibrational mode (i.e., the 7th normal mode) of CASTOR1, which showed the clearest inter-domain movement between NTD and CTD correlated with the burying and exposing of the GATOR2-binding residues, was selected for analysis. The NTD and the CTD domains of CASTOR1 are colored in pink and green, respectively. The GATOR2-binding residues, Tyr118/Gln119/Asp121, are colored in yellow.
