# Supplementary figures and images for: Structural mechanism for the arginine sensing and regulation of CASTOR1 in the mTORC1 signaling pathway
Source: Cell Discov. 2016 Dec 27;2:16051–. doi: 10.1038/celldisc.2016.51 (PMC5187391; doi:10.1038/celldisc.2016.51)

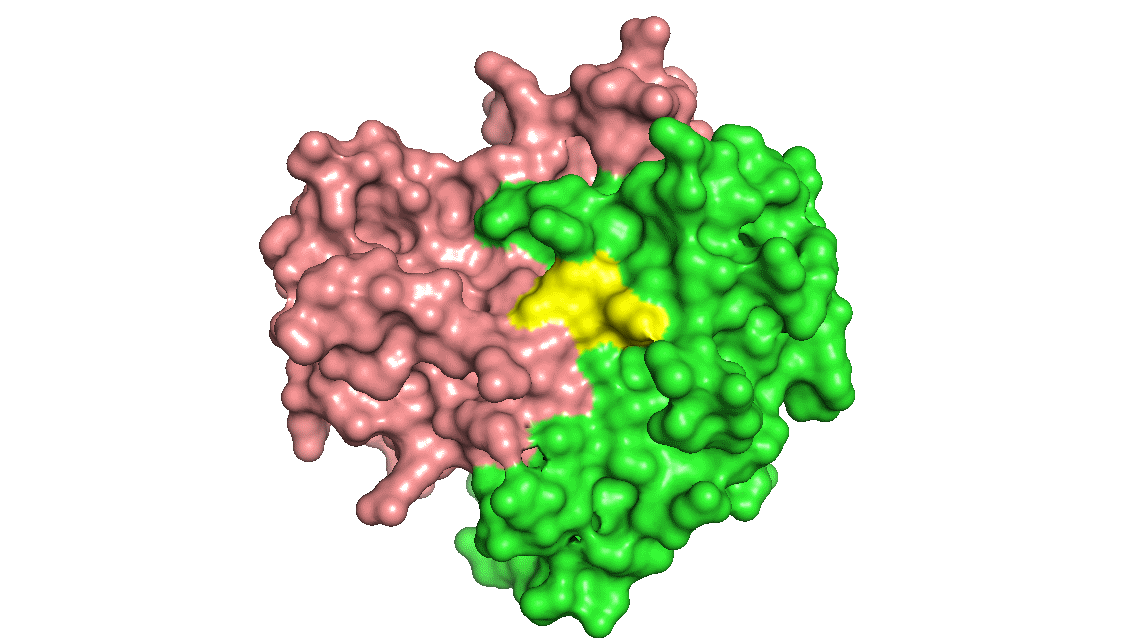

Supplement: Supplementary Video S1 [file celldisc201651-s2.gif]
